# Early Diagnosis of Anal Canal Duplication: The Importance of a Physical Examination

**DOI:** 10.7759/cureus.25040

**Published:** 2022-05-16

**Authors:** Ioannis Karamatzanis, Panagiota Kosmidou, Stavros Harmanis, Ioannis Karamatzanis, Giorgos Harmanis

**Affiliations:** 1 Internal Medicine, University of Nicosia Medical School, Nicosia, CYP; 2 Otolaryngology - Head and Neck Surgery, Mediterranean Hospital of Cyprus, Limassol, CYP; 3 Otolaryngology - Head and Neck Surgery, University of Patras, Medical School, Patras, GRC; 4 Internal Medicine, Medical School of National and Kapodistrian University of Athens, Athens, GRC; 5 Pediatrics and Neonatology, Mitera Hospital, Athens, GRC; 6 Pediatric Surgery, Mitera Hospital, Athens, GRC

**Keywords:** neonatal malformation, ano rectal diseases, anorectal malformation, congenital disease, anal canal duplication

## Abstract

Anal canal duplication (ACD) is an extremely rare congenital anomaly of the intestinal tract that presents as an extra opening of the anal canal without communication with the anorectum. We present the case of a five-year-old male presenting to the pediatrician without symptoms and upon physical examination, a duplicated anal canal along the midline was discovered. The patient was admitted for surgery and the canal was removed via mucosal stripping. Postoperatively, the patient recovered well. The present study aims to expand on our knowledge of a very rare pathological entity and emphasize the importance of a complete pediatric physical examination.

## Introduction

Anal canal duplication (ACD) is an extremely rare congenital anomaly of the intestinal tract. ACD presents as an extra opening of the anal canal without communication with the anorectum and is usually tubular [1]. It is more common in females, and it is usually diagnosed and treated in childhood and only a few cases remain undiagnosed until adulthood [2]. The extra perineal opening is most commonly found at six o’clock relative to the position of the anus in the lithotomy position [3]. The two hypotheses for the etiology of ACD are the duplication of the dorsal cloaca in early embryonal development and the recanalization of the excess dorsal cloacal membrane after normal development of the external anal sphincter [4,5]. Histological assessment for the diagnosis of ACD is composed of three types of epithelia: squamous, transitional, and columnar [3]. Anorectal fistula and rectal duplication must also be differentiated from ACD using histopathologic biopsies [3]. Constipation and pain are the most common symptoms; however, this disease most frequently presents without symptoms. [6,7].

We present the case of an asymptomatic child with anal canal duplication where we performed a complete excision of the ACD. This study aims to expand on our knowledge of a very rare pathological entity and emphasize the importance of a complete physical examination in this anatomical area.

## Case presentation

**Figure 1:**
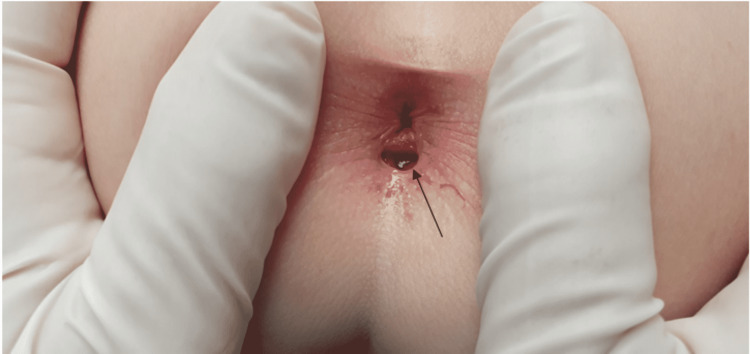
Picture of the anal canal duplication (black arrow).

A five-year-old male presented to the pediatric ward with his mother for the first time. The main reason for the visit was, as the mother revealed, that she noticed an extra opening canal in the perineal area a few days prior. The patient was asymptomatic, and his medical history was unremarkable. During the physical examination, no associated pathologies were identified but upon inspection of the perineal area, it was found that the patient had a suspected extra anal canal (Figure 1). The suspected diagnosis was anal canal duplication, and the patient was referred to a pediatric surgeon. Upon further investigation, the extra anal canal was found to be an isolated finding, not part of a syndrome.

**Figure 2:**
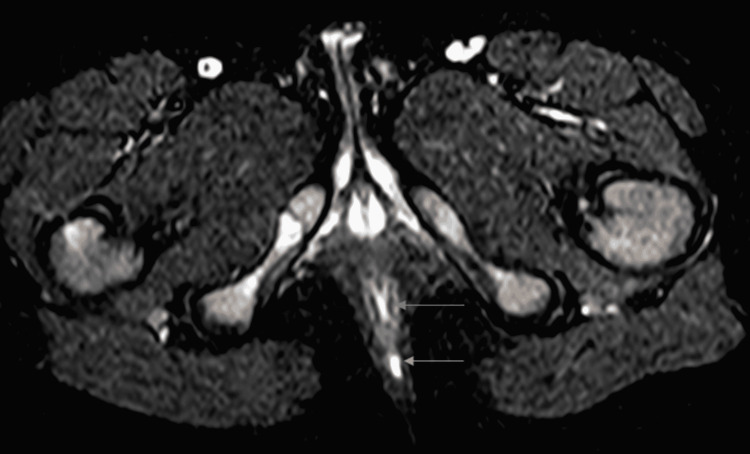
Axial MRI view of the pelvis, showing the true anal canal (blue arrow) and the duplicated anal canal (orange arrow).

**Figure 3:**
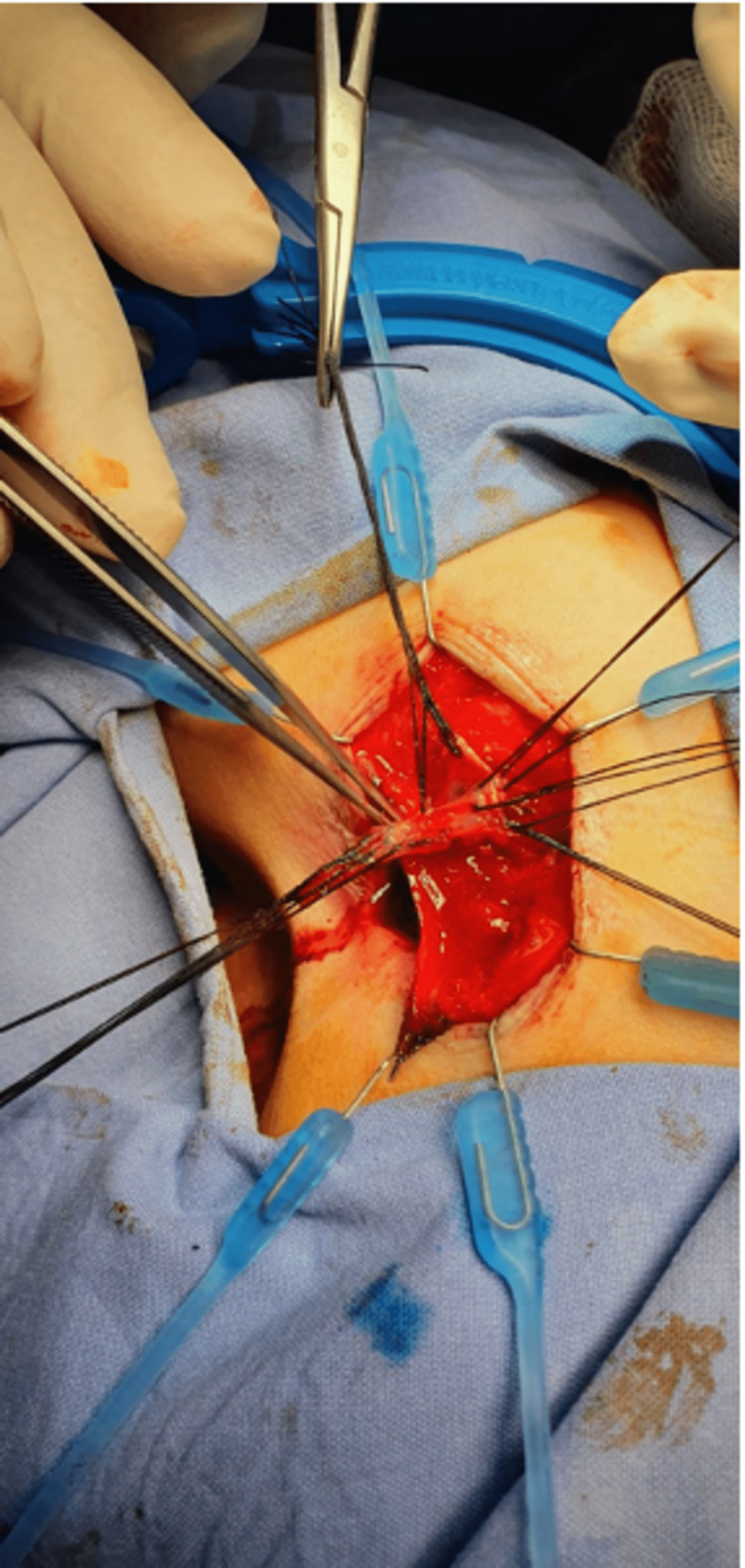
The duplicated anal canal was removed with incision and multiple silk traction sutures were placed.

**Figure 4:**
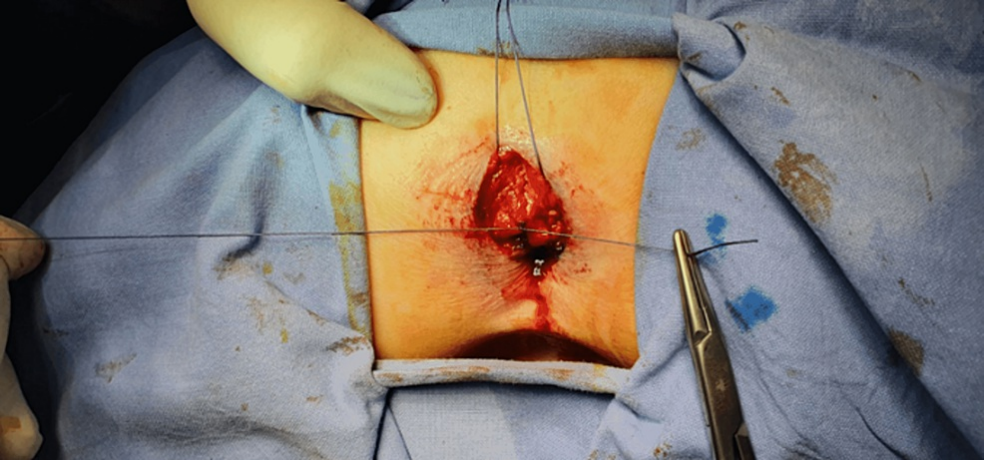
Intra-operative suturing of the removed lesion.

The patient was referred to a pediatric surgeon, who was responsible for the treatment of this infrequent pathology. Pre-surgery, an MRI was performed (Figure 2). The MRI revealed a tract measuring 5 cm in length, positioned posterior to the anal canal without communication between them as a muscular layer separated them. Following that, the patient was admitted for surgery under general anesthesia. The duplicated anal canal was excised via mucosal stripping using a posterior sagittal approach (Figure 3), biopsies were taken, and the skin was sutured (Figure 4).

**Figure 5:**
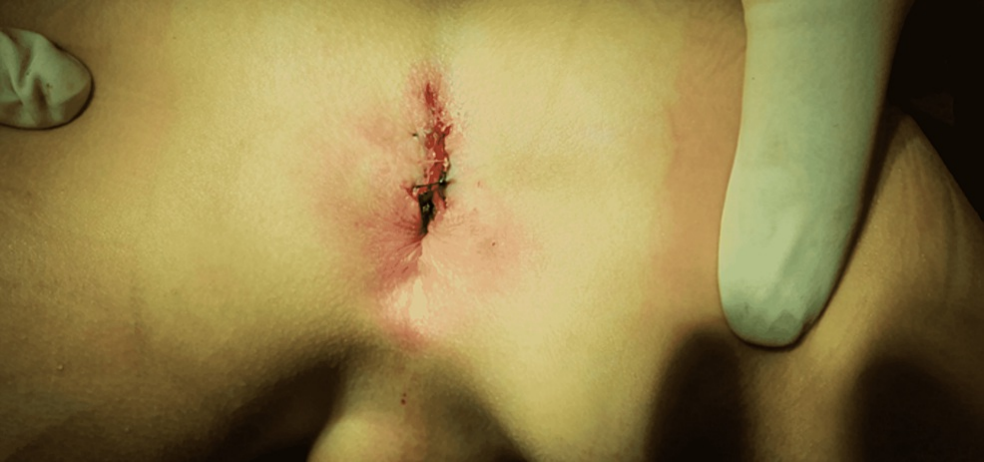
Post-surgical image of the successful repair.

**Figure 6:**
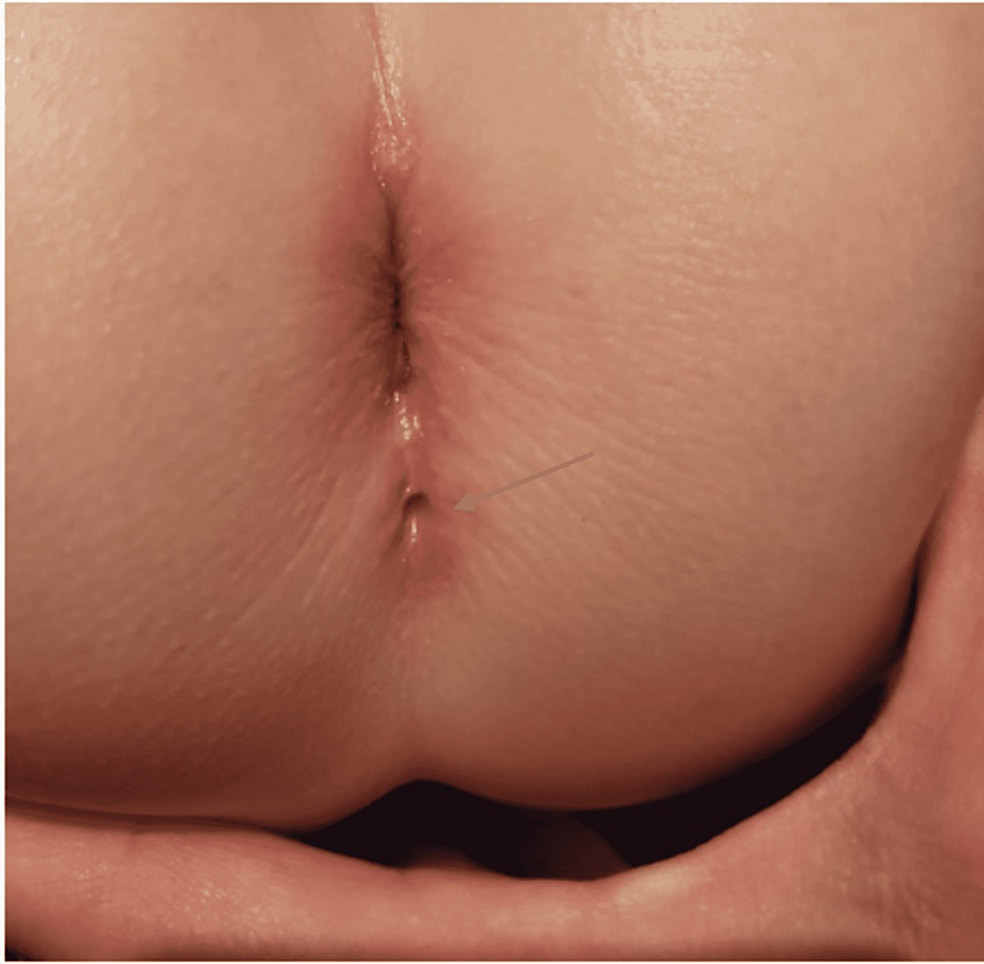
Successful repair of the anal canal duplication 9-month postoperative (blue arrow).

Postoperatively, the duplicated anal canal repair was removed (Figure 5) and the patient healed uneventfully. The patient was hospitalized for one day and was discharged. Finally, nine months after the surgery, the repair was successful (Figure 6). 

**Figure 7:**
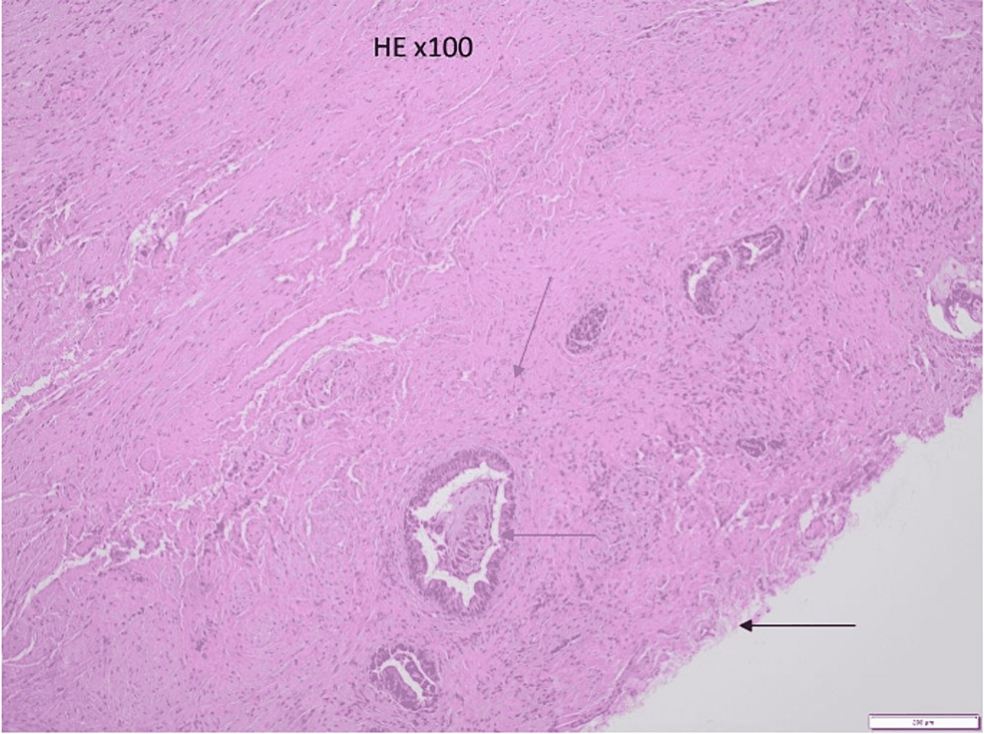
Transitional-cell epithelium (black arrow), presence of smooth muscle cells (orange arrow), and anal glands (blue arrow).

Histological examination of the specimen revealed the presence of transitional-cell epithelium, glandular tissue, and smooth muscle cells. (Figure 7). The above confirmed the diagnosis of ACD. 

Post-operative follow-up of the patient at one and two weeks showed no further complications, and the patient was pain-free. There was no evidence of recurrence until follow-up after nine months.

## Discussion

Anal canal duplication is the rarest anomaly of the alimentary tract. The condition was first described by Dukes et al. in 1956 [8]. It affects females more than males in a 9:1 ratio and is associated with another anomaly 36% of the time [9]. Currently, there are approximately 100 reported cases of ACD in English literature. 

The largest and most comprehensive literature review of ACD conducted by Trecartin et al. in 2019 found that only 38% of cases were asymptomatic, a novel finding since previous literature stated that most patients presented without symptoms [9,10]. The most common symptoms were constipation, perianal pain, and pruritus [9]. It is important to note that symptoms are less likely to occur if the patient has an isolated case of ACD [11]. The review found that age increases the probability of developing symptoms and complications [6]. Thus, early diagnosis and treatment are vital to improving disease outcomes.

A key aspect underemphasized in literature is the importance of a physical examination. Examining the patient is the primary way of identifying this condition. Diagnosis can be made by a simple perineal inspection revealing a small opening behind the anus. It was found that the lack of literature on anal canal duplication and their low frequency worldwide can lead to more missed diagnoses and, as a result, delay in treatment. Furthermore, two conditions that present with common symptoms and are found in similar locations are rectal duplication and anorectal fistulas [12]. However, rectal duplications are situated in the front or on the rear side of the anorectum. They are frequently associated with a cystic mass, whereas anorectal fistulas are not located in the posterior midline [10]. Imaging assessment confirms the size, possible communication, and mass effect of the ACD [2].

The only curative measure for ACDs is surgery [13]. Total removal of the duplicated canal or mucosal stripping is recommended to prevent further complications such as infection or malignant changes [3]. Asymptomatic ACDs can remain under observation and according to Akova et al., two patients in their study that refused surgery did not have any symptoms [6]. Prognosis after removal is favorable [11]. However, it is important to note clinicians must take additional measures if ACD presents as a syndrome, such as Currarino syndrome, or is associated with other malformations [13,14].

The diagnosis of ACD relies on two factors: clinical and histopathological assessments. ACD frequently presents with the histological findings of the squamous epithelium at the caudal end, a transitional epithelium at the cranial end, and smooth muscle cells in the wall of the lesion [3]. Thus, histopathology is important in the diagnosis of ACD.

Finally, the report of these cases in the literature is significant due to the lack of knowledge of this condition. The delay in diagnosis of children until the age of five, as in our case, reveals the need to raise awareness of this condition. This case report emphasizes the importance of the pediatric physical examination.

## Conclusions

In conclusion, ACD is the rarest congenital anomaly of the intestinal tract. The low prevalence of the disease and its asymptomatic presentation lead to missed diagnoses and delays in treatment. The gold standard for the management of this condition is the complete pediatric clinical examination, in all anatomic areas of the neonates, from birth until early adulthood, to identify and immediately treat this rare condition.
